# Research Progress of Nucleic Acid Detection Technology for Genetically Modified Maize

**DOI:** 10.3390/ijms241512247

**Published:** 2023-07-31

**Authors:** Tongyun Luo, Lujing Li, Shirui Wang, Nan Cheng

**Affiliations:** 1 College of Food Science and Nutritional Engineering, China Agricultural University, Beijing 100083, China; lliliyun@163.com (T.L.); lilujing0526@cau.edu.cn (L.L.); wangshirui@cau.edu.cn (S.W.); 2 Beijing Laboratory for Food Quality and Safety, College of Food Science and Nutritional Engineering, China Agricultural University, Beijing 100083, China

**Keywords:** genetically modified maize, amplification technology, nucleic acid detection, sensor

## Abstract

Genetically modified (GM) maize is one of the earliest GM crops to have achieved large-scale commercial cultivation globally, and it is of great significance to excel in the development and implementation of safety policy regarding GM, and in its technical oversight. This article describes the general situation regarding genetically modified maize, including its varieties, applications, relevant laws and regulations, and so on. From a technical point of view, we summarize and critically analyze the existing methods for detecting nucleic acid levels in genetically modified maize. The nucleic acid extraction technology used for maize is explained, and the introduction of traditional detection techniques, which cover variable-temperature and isothermal amplification detection technology and gene chip technology, applications in maize are described. Moreover, new technologies are proposed, with special attention paid to nucleic acid detection methods using sensors. Finally, we review the current limitations and challenges of GM maize nucleic acid testing and share our vision for the future direction of this field.

## 1. Introduction

Maize is important in the food industry because of its nutritional characteristics and wide applications in the food industry. At the same time, maize is the food crop with the largest production volume (tons) in the world [1], and, thus, it is considered to be a strategic crop for national food security by many countries [2]. Therefore, the continuous demand for maize products has driven an increase in its production. Transgenic technology can be used to change the genetic traits of maize, and many trait transformation methods are commonly used [3,4,5,6]. At present, the commonly studied traits include insect pest and herbicide resistance. A summary of the GM maize varieties approved by government authorities for release into agricultural ecosystems is shown in Table 1.

In 1996, the United States first approved the commercialization of Cry1Ab GM maize (“Bt176”, “MON810” and “Bt11”), with GM maize now having been promoted and applied for 27 years [7]. As of this publication, 244 GM maize varieties have been approved for cultivation [8]. According to a report by the International Service for the Application of Agricultural Biotechnology (ISAAA), GM crops are grown in 29 countries as of 2019. The planting area of GM maize was 6.09 × 10^7^ hm^2^ in 2019, accounting for 32% of the total planting area of GM crops. The planting area was the largest in the United States at 3.317 × 10^7^ hm^2^, followed by Brazil and Argentina [9]. The main use of GM maize globally is used for animal feed [10,11] or as an industrial raw material to extract alcohol. Only a small proportion is consumed directly by humans. The GM maize used for food is mainly used to extract maize oil [12], to make maize syrup, maize flour, or other maize ingredients [13], especially maize starch, which is widely used as a thickener, gelling agent, filler, and water-retention agent in the food industry [14]. GM maize used for food is also directly used as a raw material for food production and processing, such as in the production of tortillas in Mexico [15]; white maize in South Africa, where it is a staple food for most people [16], and maize flakes, popcorn, and maize-related snacks [17]. There is no doubt that GM maize is integrated into the lives of humans worldwide and it is, therefore, important to effectively regulate its production and processing.

The European Union (EU) has adopted a traceability management system and a mandatory labeling system for GM products in the market. The EU Regulation 1830/ 2003/EC, which came into effect in April 2004, stipulates that products containing more than 0.9% (mass fraction, % m/m) must be labeled with the words “genetic improvement” or “processed from a GM crop”. Moreover, the ratio of the DNA copy number should be used as the expression method. The measurement result of the standard substance, based on the mass percentage, shall prevail, and the measurement result of the copy number percentage (DNA copy number ratio, % cpT/cpE) shall be converted into a mass percentage [18]. In China [19], North America [20,21], Australia, New Zealand [22], India [23], and other countries, there are laws and regulations to clarify the labeling management system for GM products. This ensures that business operators and consumers have access to accurate information, so that they can effectively exercise their freedom of choice and be able to control and verify label claims. The latter requirement makes it a necessity to detect the presence of GM organisms (GMOs) through reliable detection methods. The detection of GM crops can be roughly divided into the detection of nucleic acids, proteins, and metabolites, according to the target. In biological cells, DNA is relatively stable compared to proteins, and is not easily destroyed, even after the crops are processed, such that trace or detectable amounts of DNA fragments may remain in the product. Therefore, DNA detection is often the preferred method for identifying GM components of crops. In common polynucleotide detection methods, targets such as promoters (e.g., *Cauliflower mosaic virus* (CaMV) 35S promoter [24]), terminators (e.g., the nopaline synthase terminator (*T-nos*) [25,26]), or marker genes (*CP4-EPSPS* and *pat* [27,28]) are usually invoked as surrogate markers for transformation. However, methods for testing the above-mentioned target genes cannot distinguish between different strains of GM crops with the same exogenous gene transferred, and the specificity is low. Therefore, a pair of primers spanning the junction of the inserted transgene and the flanking DNA is often used to identify transgene-specific events [29,30], that is, the strain of the GM crop, by detecting the connecting region of the foreign gene and the plant genome. At present, this method is widely used to identify GM maize lines, such as MON810, NK603 [31], Bt11, TC1507, GA21 [32], and so on.

Generally, nucleic acid detection needs to go through a process of nucleic acid extraction and purification, and target detection to obtain results. The purity and quality of the extracted nucleic acid determine the effectiveness of the subsequent amplification, and the setting of the target is the basis for the specificity of detecting the GM maize sample. The method of obtaining the result is also based on intuition and convenience, according to the needs of different detection scenarios. For example, simple and rapid on-site testing methods are crucial for regulating the import and export of GM crops. In this context, the manner in which representative samples are collected, and the timing and reliability of the analysis, is critical for the smooth implementation of regulations and market surveillance.

## 2. Nucleic Acid Extraction Technology

**Figure 1:**
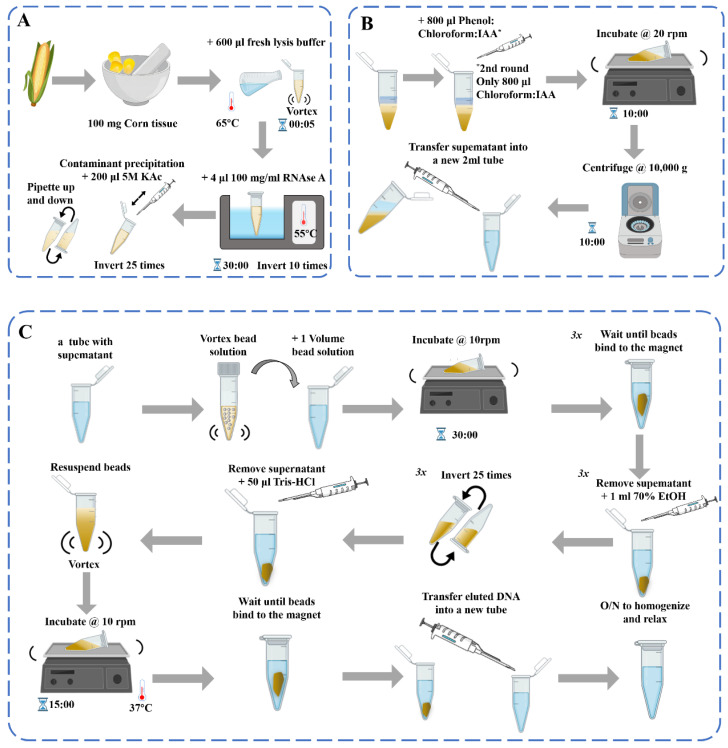
Schematic diagram of the DNA extraction process [39]: (**A**) DNA extraction; (**B**) Contaminant precipitation and Phenol: Chloroform purification 2×; (**C**) Genomic DNA purification with beads.

DNA-based assays are used to examine the transgenic status of processed products, but the quality of the DNA must be ensured. In the study of molecular biology detection methods of different kinds of materials, the choice of DNA extraction methods directly affects its performance and utility. At present, the common extraction methods of plant genomic DNA are sodium dodecyl sulfate (SDS) [33], cetyl trimethylammonium bromide (CTAB) [34], urea extraction method, Chelex-100 method [35], alkali lysis method, polyvinyl pyrrolidone-40 (PVP-40) [36], high-salt and low-pH extraction method [37], and commercial kits [38], etc. These DNA extraction methods are generally consistent in principle, involving cell lysis for DNA release and the removal of impurities for DNA purification (Figure 1). However, the content of proteins, polysaccharides, phenols, and other substances in different plant materials is not the same, which causes great difficulties in the extraction, separation, and purification of DNA. For plant-derived samples, the appropriate method should be selected according to the characteristics of the sample, and the methods may need to be adjusted and optimized.

In the process of DNA extraction of maize samples, there are related extraction methods of maize raw materials for various plant tissues, such as pollen, leaves, maize silk [40], endosperm, and others [41]. Maize is an angiosperm, and its fruit has a unique structure, mainly composed of a pericarp, seed coat, endosperm, and embryo (germ, radicle, hypocotyl, and cotyledon). The pericarp and seed coat of sexually reproduced maize are developed from the ovary wall and the integument of the female parent, and all of the genetic material comes from the female parent. The pericarp and seed coat of mature maize almost grow together and are difficult to separate. The endosperm develops from the fertilized polar nucleus, and its genetic material is different from that of the embryo and adult plant; therefore, proper consideration should be given when extracting raw materials. The traditional gene-screening method involves extracting genomic DNA from the leaves [42,43] (or young leaves) of individual plants after planting large populations of maize in the field. The single-seed maize DNA extraction method mainly involves crushing the seeds or using the seed endosperm to extract DNA. DNA extraction methods for crude maize products are mainly used in the detection of GMOs in feed [44,45]. According to the degree of DNA degradation from smallest to greatest, the relevant foods can be divided into raw maize, frozen maize, canned maize, and dry packet maize samples [46]. The deep-processed corn products are mainly seasoning, puffed, fried, saccharified, and fermented flour products, such as maize oil [47], maize starch, maize chip, popcorn, maize stick, crisp maize horn, wowtou [48], etc. 

Cell lysis, including mechanical, enzymatic, and chemical lysis, is an essential process for DNA extraction from cell tissues. Mechanical cracking, such as grinding in liquid nitrogen, heat shock, homogenization, and ultrasonic treatment [49], may lead to DNA breakage, which generally plays an auxiliary role in practical applications. The ultrasonic intensity and gap time need to be strictly controlled to avoid excessive DNA breakage. Enzymatic cleavage uses specific enzymes (such as pectinases) to destroy cells and release nucleic acids that can be extracted in conjunction with chemical cleavage. 

CTAB and SDS are the two most commonly used and effective chemical lysing agents. In general, surfactants such as CTAB and SDS tend to interact with polymers (e.g., proteins and DNA) driven by electrostatic, diaxial, and hydrophobic forces [50]. CTAB can dissolve cell membranes, and in high-salt (>0.7 mol/L NaCl) solutions, CTAB can form soluble and stable complexes with proteins and polysaccharides, but cannot precipitate nucleic acids [51]. In low-salt (0.1~0.5 mol/L NaCl) solutions [52], SDS can, under alkaline conditions of 55~65 °C, lyse cells and make DNA free, while denaturing proteins and binding to them [53]. Recently, ionic liquids (ILs) and magnetic ionic liquids (MIL) have been explored as novel solvents for extracting DNA from complex biological matrices [54]. ILs and MILs promote DNA extraction through electrostatic interactions between cationic and negatively charged phosphate backbones, and hydrophobic interactions between the alkyl chains of the solvent and DNA bases [55,56]. Microscale electroporation is an emerging technology for the release of intracellular materials [57]. Its mechanism of action is that, when the electric field intensity of the applied electric pulse reaches a certain order of magnitude, the cell membrane undergoes a configurational change, and a large number of micropores appear. This increases the permeability of the cell membrane, which is conducive to the release of various macromolecular substances (such as DNA, RNA, proteins, chemical small molecules, etc.). However, adjusting the applied voltage and pulse length to determine the optimal conditions for cell lysis and DNA extraction still requires experimental verification.

Crude DNA extracts contain large amounts of protein, RNA, sugars, and other impurities; therefore, DNA purification is essential. Most proteins can be removed by denaturation and precipitation after treatment with chloroform or phenol, which are common methods of protein removal [58]. The alternating use of phenol and chloroform, two different protein denaturants, can enhance the effect of protein removal [49]. It should be noted that these chemicals, phenol and chloroform, have certain oxidizing properties and can seriously damage DNA if used improperly. For example, guanine is particularly sensitive to oxidation, and exposure to phenol/chloroform can result in the formation of 8-oxoguanine [59]. Furthermore, phenol and chloroform are volatile and toxic, with chloroform classified as “reasonably expected to be a human carcinogen based on sufficient evidence of carcinogenicity in laboratory animal studies”, according to a U.S. Department of Health and Human Services report on carcinogens [60]. 

RNA can be removed by digestion with RNase A for 1~2 h at approximately 37 °C, or DNA can be purified by cesium chloride density gradient centrifugation, which results in a high-quality DNA preparation [58]. Maize plant cells have thickened secondary walls and large vacuoles that store a large number of secondary substances, such as polysaccharides and polyphenols. The surfactant CTAB is better than SDS at removing polysaccharides [61,62]. Appropriately increasing the content of CTAB (according to the actual situation, such as increasing it to 3%, but not using less than 1% [63]) β-mercaptoethanol (0.2%–1%, determined according to the actual situation) [64] can effectively remove polysaccharides and other secondary biomolecules. Simultaneously, the polyphenols contained in plants are oxidized under the catalytic action of polyphenol oxidase, resulting in a lower quality of the extracted DNA. The main methods to remove the effects of polyphenols include adding antioxidants to the extraction medium or adding PVP or ascorbic acid during grinding [65]. Further oxidation of phenolic compounds can be prevented by using a higher concentration of salt and a less-acidic medium [66]. Commercial solid-phase extraction kits with silica-based centrifugal columns have been developed to standardize procedures and make them more efficient. These kits use cleavage buffers containing CTAB or SDS, binding buffers consisting of dissociative salts to facilitate DNA adsorption onto silica adsorbents, and washing buffers containing organic solvents to eluate and purify the DNA [67]. The entire extraction process is conducted at room temperature, the rigor of the experimental requirements is low, and the concentration and purity of the DNA obtained meet the requirements of most contemporary molecular biology applications. Currently, commercial kits used for plant DNA extraction also employ functionalized magnetic materials to simplify the purification step through the use of external magnets. 

For DNA extraction of GM maize, the traditional CTAB method is the most suitable method for extracting amplifiable DNA from highly processed maize gluten, which is often used as a protein-rich feed ingredient. This method can produce sufficient amounts of amplified DNA in laboratory tests to control the compliance of the tested substrate with event tolerance limits and labeling thresholds for authorized GM maize [44]. The SDS extraction method often results in a higher DNA yield, better cell lysis efficiency, a lower DNA shear rate, and higher diversity than the CTAB method [68]. Therefore, among the many DNA extraction methods at present, new improvements of both the CTAB and SDS methods are currently under development [53,69].

With rapid developments in the field of molecular biology, precision diagnosis, and treatment, the demand for emerging nucleic acid extraction technologies with high throughput, purity, and quality is constantly increasing. As a simple, fast, reliable and automated nucleic acid extraction method, the magnetic beads(MBs) method [70] for nucleic acid extraction has attracted more and more attention. The process of DNA extraction by the MBs method is simple, without repeated centrifugation, column separation, or vacuum filtration [71]. The entire process consists of four steps including lysis, binding, separation, and elution. Therefore, the process is fast and the extraction efficiency is high, culminating in extracted nucleic acids that are high in purity and concentration. Moreover, it is safe and non-toxic, does not use toxic reagents (such as phenol, etc.), reduces personnel hazards, and can be easily adapted for automated batch operation [72]. MBs and automatic nucleic acid extraction instruments can be used for high-throughput automated extraction of nucleic acids from large numbers of clinical samples. MBs with functionalized surfaces that can capture nucleic acids have been widely used to extract nucleic acids from biological samples, and multiple forms have been developed [73]. Examples include manual extraction using magnetic frames or microfluidic chips [74], automated robotic processing, and also the separation of ctDNA by superparamagnetic bead particles in microfluidic platforms for early cancer detection, etc. Combining them with traditional gene extraction methods can efficiently and quickly extract plant nucleic acids, and has an absolute advantage over other methods in the detection of low-content genetically modified components. Sebastian et al. [75] used centrifugal microfluidic technology, using continuous rotating magnetophoresis to facilitate magnetic bead integration and nucleic acid extraction where needed. This solution solves the drawbacks of magnetic bead-based solid-phase extraction that may cause nucleic acid loss due to the handling of magnetic beads when they are transferred from one chamber to another, resulting in higher yield and purity in the end. Jiang et al. [70] and others established a no-elution MB-based nucleic acid extraction method by introducing PEPPG F68 into the lysate and using NaOH solution instead of alcohol as the washing buffer. It avoids the dilution and loss of the target nucleic acid during the elution process, as well as the possible loss of sensitivity and false-negative results. At the same time, the detection sensitivity of loop-mediated isothermal amplification (LAMP) is significantly improved, which has broad application prospects. The current demand by molecular biologists is for convenient, rapid, and inexpensive DNA extraction and detection methods and more compact, portable equipment options to enhance real-time capabilities. The merging of extraction and microflow body technologies [76] to automate nucleic acid detection [77,78] is also in high demand.

## 3. Traditional Detection Technology

### 3.1. Variable-Temperature Amplification

Event-specific polymerase chain reaction (PCR) targeting unique sequences spanning the insert DNA and flanking genomic DNA has the highest level of specificity and is commonly used to confirm the identity and authorization status of GMO ingredients and to quantify GMO content [79]. DNA-based PCR methods are considered the most reliable and versatile techniques for the identification and quantification of GMOs, with the chief method being variable-temperature amplification. Variable-temperature amplification technology includes three steps, high-temperature denaturation, low-temperature annealing, and a suitable-temperature extension, and generally refers to PCR technology and its derivatives, such as real-time fluorescent PCR [80,81], droplet digital PCR (ddPCR) [82], nested or semi-nested PCR [83], multiplex PCR [84], etc.

The PCR method is the most commonly used molecular detection technique and is the standard method for detecting GMOs [85]. Standard qualitative PCR is the most commonly used PCR detection method. The mechanism is as follows: after the primer and template DNA are specifically combined in accordance with the principle of complementary base pairing, under the catalysis of Taq DNA polymerase, using deoxyribonucleotide triphosphates (dNTPs) as the raw material, a new DNA strand is synthesized according to the principle of semi-conservative replication. After “n” times of amplification, the total number of progeny DNA is 2^n^, and finally achieves a million-fold amplification of the number of target DNA fragments, which facilitates the detection of subsequent target DNA fragments [86] and, finally, achieves a million-fold amplification of the number of target DNA fragments, which facilitates the detection of subsequent target DNA fragments. Standard PCR is used for the amplification of transgenic crop genes owing to its simple operation, high efficiency, and low cost. Quantitative PCR (qPCR), based on a standard curve, is considered the gold standard technique for the analysis of GMOs because of its high sensitivity and good stability [87]. Real-time fluorescent qPCR [88] is a technology that adds fluorescent groups to the PCR reaction system and uses the accumulation of fluorescent signals to monitor the entire PCR reaction process in real time. This technology uses the strength of the fluorescent signal to determine the number of specific amplification products over time, and an unknown template is quantitatively analyzed using a standard curve. This technology can quantitatively analyze DNA templates and has the characteristics of high sensitivity, specificity, and reliability; low pollution; and timely and accurate detection. It can perform both absolute and relative quantifications and is widely used to inspect GM maize [89]. Various forms of qPCR are constantly being developed to meet the needs of practical applications, focusing on duplex and multiplex reactions to improve detection throughput and efficiency.

The concept of digital PCR (dPCR) was first proposed by Vogelstein et al. in 1999 [90]. In dPCR, the reaction mixture is divided into many individual reactions called partitions, and each reaction does not contain one or more copies of the target. Reads are partitioned as negative or positive at the endpoints, and DNA concentrations are calculated using a Poisson distribution [91]. The partitioning of reaction volumes using wells on a chip in microfluidics/chip-based dPCR [92] and droplets in emulsion/ddPCR [93] are the two main approaches. In cdPCR, reactions are divided into hundreds or thousands of chambers in a single plate or array. Many studies have used chip-based platforms, such as the microwell chip-based QuantStudio 12k flex dPCR and 3D dPCR (Life Technologies), for the detection of GMOs in the field [94,95]. The Constellation system (Formulatrix) is a plate-based microfluidic dPCR system that offers five-color multiplexing. The biggest difference between the cdPCR platforms is the number of partitions created per sample and the number of samples analyzed in one run [96]. ddPCR has a synergistic effect on droplet microfluidics. It improves the sensitivity of PCR at the single-molecule level by dividing tens of microliters of PCR mixture into tens of thousands of droplets and it can perform absolute quantification without a standard curve, thus avoiding the amplification efficiency bias observed in qPCR. It enables accurate target determination even at low copy numbers and can be significantly cost-effective when combined with multiplexing [97]. However, the droplet reaction generator used for ddPCR is bulky and complicated, which is an important limitation for its use in on-site detection. Thus far, microfluidic platforms for droplet generation using centrifugal forces, such as those utilizing ferrofluids, electromagnets [97], and surface acoustic waves [98]. Using the working principle of the indirect pressurization method, Park et al. [99] developed a pushbutton-activated microfluidic dropenser (droplet dispenser). Its use for sample preparation in ddPCR eliminates the need for benchtop droplet generators and automated pipetting, making ddPCR an on-site molecular diagnostic tool. In conclusion, dPCR has proven to be an effective tool for the quantification of maize and soybean GMOs and GMOs in complex matrix samples with precision and accuracy the same as or better than qPCR methods [100]. Recently developed multiplex dPCR methods [87,101] may be useful for analyzing samples containing multiple genetic modification events.

More types of PCR technologies are constantly being developed to meet the requirements for on-site rapid detection and high throughput. The use of an ultrafast PCR system can significantly reduce PCR run times and the number of reagents required for analysis. Therefore, ultrafast PCR systems have recently been studied and applied in various fields. The latest example of an ultrafast PCR system is a system used in rice detection [102]. The analysis principle is the same as that of real-time fluorescent qPCR, based on SYBR green [103], except that Evagreen dye is used as the intercalating dye instead of SYBR green. It requires 18% of the detection time of traditional PCR and 23% of the detection time of real-time PCR, and it can support small portable analyzers, thus providing a new strategy for the on-site detection of GM maize.

### 3.2. Isothermal Amplification

Isothermal nucleic acid amplification technology is used for nucleic acid amplification at a constant temperature. According to the different methods of single-stranded template formation, isothermal amplification can be divided into the following four categories: (1) strand-displacing DNA polymerase-mediated reactions such as Loop-Mediated Isothermal Amplification (LAMP) [104], Rolling Circle Amplification (RCA) [105], Cross-Primed Amplification (CPA) [106], Nucleic Acid Sequence-Based Amplification (NASBA [107]), and Multiple Displacement Amplification (MDA) [108]; (2) Enzymatic unwinding primer annealing reaction, such as Helicase-Dependent Amplification (HDA) [109], Recombinase Polymerase Amplification (RPA) [110], and Ligase Chain Reaction (LCR) [111]; (3) RNA transcription-based amplification, such as Transcription Mediated Amplification (TMA); and (4) Requires reactions assisted by single-strand cleavage enzymes, such as Strand Displacement Amplification (SDA) [112], and Isothermal Strand Displacement Amplification (iSDA) [113]. LAMP, RPA, and CPA are widely used for the detection of GM crops.

LAMP employs a DNA polymerase and a set of four specially designed primers that recognize a total of six different sequences in the target DNA. Internal primers containing the sequences of the sense and antisense strands of the target DNA initiate LAMP [104]. The product is a mixture of stem-loop DNA with stems of various sizes and cauliflower-like structures. Multiple loops are induced by annealing between alternating inverted repeats of the target sequence in the same strand. This enables simpler and more selective detection. For example, through a mechanism similar to multivalent antigen antibody interactions, the target sequence has a higher degree of specificity [104]. This method is insensitive to inhibitors, and can be used with crude DNA samples. LAMP is a simple and reliable GM detection method that can be performed on a thermal cycler, heating block, or portable constant temperature real-time amplification system. After the reaction is completed, using nucleic acid staining or fluorescent dyes, such as SYBR® Green and hydroxynaphthol blue, the LAMP products can be visualized and monitored using turbidity analysis or real-time LAMP [114]. Since the first report on the LAMP method in 2000, the number of studies using LAMP to detect GM ingredients has increased annually [115,116,117].

**Figure 2:**
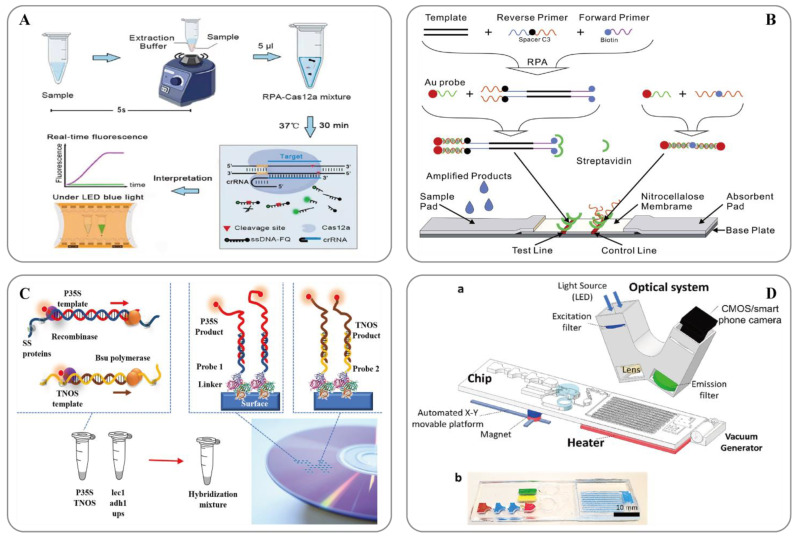
(**A**) Schematic showing the ORCD assay system for rapid and visual nucleic acid detection [123]; (**B**) Schematic of the LFNAA (lateral flow nucleic acid assay) [125]; (**C**) Scheme of the assay for GMOs detection based on multiplex RPA amplification (left) and hybridization assay in the array format (right) [127]; (**D**) Microfluidic platform for sample-to-answer digital RT-RPA [126]: (a) Schematic illustration of the total system consisting of a magnetic platform, heater unit, vacuum generator, detection part with camera, and light-emitting diode (LED). The disposable chip has three operational zones to prepare and mix the reagents and detect the pathogen agent. (b) Dye-loaded chambers for visualization of chips. Brown, blue, and red show lysis, washing and elution chambers, respectively, for sample preparation. Green and yellow chambers show RPA mixture and mineral oil chambers, respectively.

RPA technology was first proposed in 2006 by Piepenburg et al. [118]. In RPA, isothermal amplification of specific DNA fragments is achieved by the binding of reverse oligonucleotide primers to the template DNA and extension via DNA polymerase. It does not require the orientation of the primers to their complementary target sequences. RPA can be used to amplify cDNA generated by the reverse transcription of double-stranded DNA, single-stranded DNA, methylated DNA, RNA, or miRNA, and multiple reverse transcriptases are used for RPA [110]. When using RPA directly in milk [119] or seed powder [120], only thermal lysis, nuclease-free water lysis, or EzWay^TM^ Direct PCR buffer are required to release the desired nucleic acid. With the assistance of a variety of enzymes, the in vitro amplification of nucleic acid can be completed at a constant temperature of 31–37 °C for 20 minutes [110]. Traditional in vitro nucleic acid amplification techniques do not have a rapid response, high sensitivity, high specificity, or low equipment dependence. Compared with SDA, RCA, and LAMP, RPA does not require an initial denaturation step to generate single-stranded (ss)DNA from double-stranded (ds)DNA targets, highlighting its suitability for use in the field [121]. In 2014, a commercial RPA kit launched by the British company, Twist DX [122], made the detection more convenient. Simultaneously, a variety of probes can be combined to expand the application range of RPA technology. RPA appears to be particularly well suited for multiplexing, where different targets can be verified with different efficiencies; however, it currently requires laborious optimization steps. Clustered regularly interspaced short palindromic repeats (CRISPR)/CRISPER-associated protein [123], lateral flow assays [124,125] and microfluidics [126] have provided additional options for the on-site point-of-care detection of transgenes (Figure 2).

CPA technology is an isothermal DNA amplification system developed by Us-Tar Biotechnology Co., Ltd [106]. The system relies on only one ring structure for replication [128]. The CPA assay enables the amplification of nucleic acid sequences at a constant temperature and requires only an enzyme with strand displacement activity and a set of five primers to perform the CPA reaction, without the need for an initial denaturation step or the addition of a nickase [129]. At an assay temperature of 63 °C, the formation of primer template hybrids under transient spontaneous denaturing bubbles in the DNA template are more favorable than the re-annealing of the template strands by high concentrations of primers relative to the template DNA. Strand displacement is facilitated by annealing cross-primers with 5’ ends that are not complementary to the template strand and the binding of displacement primers upstream of the cross-primers. The resulting exponential amplification of the target DNA is highly specific and sensitive [106]. CPA has traditionally yielded results through expensive fluorescence-based techniques, tedious gel electrophoresis procedures, or the measurement of turbidity using a spectrophotometer. These methods require complex and bulky optics or exposure to carcinogenic dyes, which limits their wider use in resource-limited laboratories [128]. In addition, colorimetric indicators such as pH-sensitive dyes (neutral red), malachite green (MG), and hydroxynaphthol blue (HNB) [130] were also used as complementary techniques to monitor CPA responses.

### 3.3. Gene Chip Technology

Gene chip technology is a GM food detection technology developed by the American company Affymetrix in the 1990s. It has rapidly developed as a high-tech molecular biology tool in recent years. Gene chips are also known as DNA chips or microarrays [131]. This technology involves arranging a large number of DNA fragments or oligonucleotide fragments at an orderly density on a solid-phase carrier, using specific probes on the surface of the solid-phase carrier to hybridize with labeled samples, and using a chip scanner to detect and analyze hybridization signals [132]. It can accurately detect different types of DNA sequences in samples qualitatively and quantitatively. When microarray hybridization is used for gene analysis, information on differences in gene expression can be obtained from very few experimental samples. When using this technology for detection, the sample is pretreated and purified to obtain a highly pure DNA sample, which is then amplified by PCR, labeled with fluorescence, and hybridized with the DNA probe on the gene chip. The signal is then read to obtain the result [133]. Gene chips can immobilize a large number of oligonucleotide probes for different target genes; therefore, gene chip technology can detect dozens or even hundreds of genes simultaneously [134] and can detect multiple components in GM foods. Lu et al. [135] used a gene chip detection method combined with multiplex PCR to simultaneously detect multiple pairs of genes on a single chip. Seven types of GM maize components were detected: Bt176, Bt11, GA21, Mon810, Mon863, TC1507, and NK603. This method greatly improves the accuracy and efficiency of detection, with a sensitivity as high as 0.01%. Turkec et al. [131] tested 1830 different probes and developed a high-density oligonucleotide microarray platform for 12 GM varieties (nine maize and three soybean varieties). This method eliminates the need for a PCR amplification step, which simplifies the analysis and allows the quantification of each detected GMO, enabling the specific detection of each GM crop with a sensitivity of 1% (DNA concentration).

Gene chip technology offers advantages, such as a high level of parallelism, high throughput, high specificity, high sensitivity, and automation. However, due to the late start of the development of this technology, strong comprehensiveness, strong professionalism, and high cost, the process of making gene chips is relatively complicated, and there are certain problems, such as background interference. Therefore, the popularization and promotion of this technology in practical applications are limited [136]. At present, the types of GM edible agricultural products grown worldwide is limited, and the detection of GM ingredients often only requires the detection of dozens of target genes, whereas gene chip technology can perform multigene or even whole-gene detection [134]. Therefore, considering the experimental cost and utilization rate, this technology is currently unsuitable for transgene detection. In general, gene chip technology requires extremely small samples and has the advantages of being fast, time-saving, pollution-free, accurate, and suitable for automated operation; however, current research using this technology is in the field of disease diagnosis and microbial detection, and research on transgenic detection is focused more on microfluidic chips [137]. Microfluidic chips integrate multiple operating platforms into one chip through micro-processing technology, with less consumption of samples and reagents, a fast reaction speed, and a large number of parallel processes. Therefore, it has greater development potential.

## 4. New Nucleic Acid Detection Technology

### 4.1. CRISPR/Cas System-Based Detection

**Figure 3:**
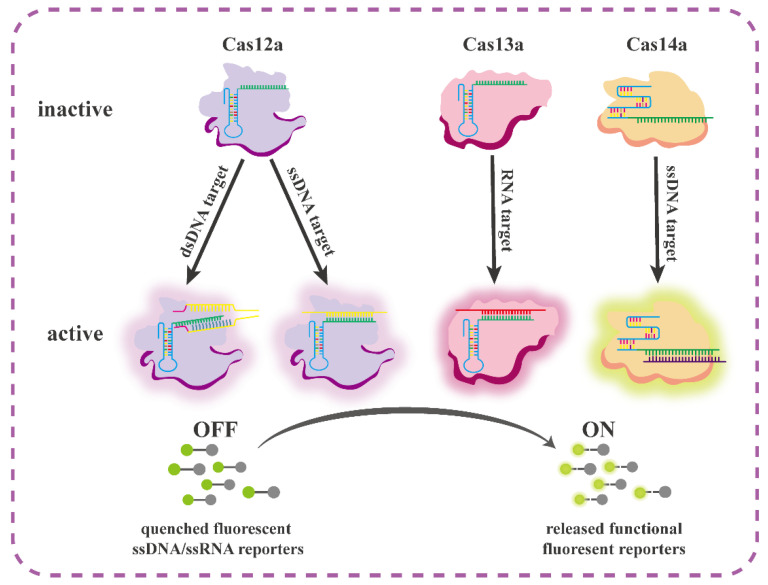
Schematic diagram of a new detection strategy based on Cas effector “incidental” cleavage [138].

With the continuous development in the fields of biological science and technology, detection technologies and methods for GM plants have also developed. New methods and technologies for the detection of GM plants are constantly emerging. New technologies for the detection of nucleic acid components in GM crops include methods based on the CRISPR/Cas system. The CRISPR/Cas system has revolutionized rapid molecular diagnostics. In this system, because of the trans-cleavage activity of the Cas protein, the target sequence only requires a short DNA fragment that can match the 20 nt sequence complementary to the CRISPR-derived RNA (crRNA) [138]. Subsequent formation of the Cas protein/crRNA/short nucleic acid complex activates the CRISPR/Cas detection system. Therefore, the CRISPR/Cas system has excellent selectivity and programmability, and can be used to develop a detection platform for short fragments of DNA. Currently, a class of systems represented by Cas2, Cas9a (Cpf12), Cas1b, Cas12a (C13c2), and Cas2a (Cas14f12) is the most studied. These systems apply single-protein effectors, and have the advantages of simple operation, high specificity, and high sensitivity. For Cas12, Cas13, and Cas14, as shown in Figure 3 [138], when the guide RNA captures the nucleic acid target, a Cas/crRNA/target ternary complex forms and activates the transcleavage activity of Cas to cleave ssDNA/ssRNA [139]. The CRISPR/Cas system can also be combined with various other detection technologies. Currently, the CRISPR/Cas12a system is the most commonly studied system. It combines PCR and fluorescence visualization detection methods [140], recombinase polymerase amplification (RPA-Cas12a-FS) [141], lateral flow strip technology [142], multiple PCRs, and CRISPR/Cas13a (MPT-Cas12a/13a) [143], and provides a new strategy for nucleic acid detection in GM maize.

### 4.2. PCR-Based High-Throughput Detection

Agarose gel electrophoresis (AGE) and qPCR are the two most commonly used methods to visualize results. However, the resolution of AGE and the number of qPCR detection channels are limited, making it difficult to detect multiple targets. Capillary electrophoresis (CE) [144], on the other hand, uses primers with fluorescent labels at the 5′ end to detect amplicons of specific lengths, with high resolution, and can easily distinguish amplification peaks that differ by only 1 bp [145]. In addition, this method can detect a wide range of fluorescent signals. In addition to its high resolution, CE is simple to perform, can simultaneously detect multiple target sequences, and is easy to automate, making it an excellent choice for high-throughput GMO inspection. Yi et al. [146] developed a GM maize detection system by combining the advantages of multiple PCRs with CE and constructed an event-specific multiplex system for 29 GMO maize events, while adding elements and gene targets, to cover more than 98% of all GMO maize events. In addition, the application of microfluidic technology provides additional possibilities, mainly for the application of microfluidic chips. Microfluidic chips integrate multiple operating platforms of conventional laboratories in a single chip through micromachining technology. This has the advantages of low sample and reagent consumption, high detection efficiency, high integration, and the performance of a large number of parallel processes simultaneously. This technology shows great development potential in the fields of biology, chemistry, and medicine. Junyi et al. [147] developed TaqMan microfluidic chip technology and applied it to a real-time fluorescent qPCR platform for the high-throughput detection of GM maize strains. This system can simultaneously identify 17 GM maize strains, realizing the parallel detection of multiple strains, the complete closure and microquantification of the amplification detection reagent system, and a reduction in the detection costs. The method provides technical support for the rapid identification and detection of hybrid strains of GM maize.

### 4.3. Technology in Combination with Biosensors

Biosensors have inherent advantages such as ease of use, automation potential, and inexpensive and integrated devices, and, therefore, they show promise for applications in areas with limited resources. Gene sensors are constructed using fixed ssDNA. They can be hybridized with complementary strands, and have high specificity, thus eliminating the limitations of the experimental conditions for DNA amplification and the inevitable false positives caused by primer dimers.

#### 4.3.1. Lateral Flow Biosensing Technology

**Figure 4:**
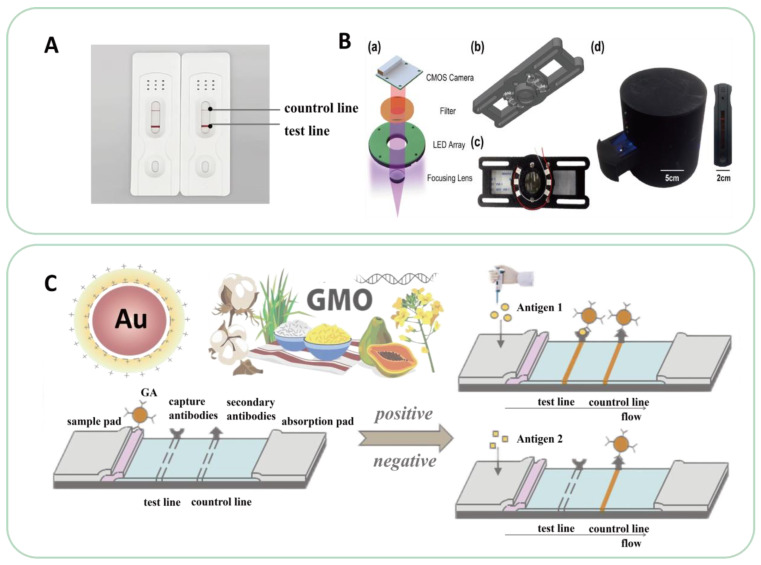
Lateral flow biosensing (LFB): (**A**) Physical diagram of Lateral Flow Biosensing; (**B**) Inner assembly of the design of the UV fluorescence-based optical reader [155]: (a) Exploded view of the optical reader. The fluorescence sensor excites the fluorescence signal with a 380 nm ultraviolet (UV) LED and captures the spectrum larger than 530 nm in wavelength with a long pass filter. The Complementary metal-oxide-semiconductor (CMOS) camera and the LED array are controlled by a Raspberry Pi inside the optical reader. The sensor holder and the Raspberry Pi are not shown in the figure for clarity. (b) Computer aided design (CAD) design for the UV fluorescence sensor coupled with other parts. (c) Assembly view of the UV fluorescence sensor coupled with other parts. (d) External view of the UV fluorescence-based optical reader with casing; (**C**) Schematic diagram of LFB.

Lateral flow biosensing is a fast, simple, and inexpensive method for the detection of GM foods, enabling the on-site analysis of a variety of samples, including water, blood, food, and environmental samples [148], as shown in Figure 4. It is also one of the most commonly used methods for rapid onsite GMO detection. Generally, the detection sensitivity of side-flow chromatography depends on the ability of GM crop samples to bind to labeled antibodies and trap antibodies fixed on the test line (T-line). Therefore, gold nanoparticles (AuNPs) and other markers (such as AuNPs/enzymes) are commonly used in nanocomposites for solution flow rate control and signal amplification. This increases the probability of binding or amplifying the colorimetric signal to improve detection sensitivity. With the development of this technology, nanoparticle-based side-flow biosensors have been widely used to analyze various substances [149,150,151].

Yi et al. [129] studied label-free cross-priming amplification coupled with a nanoparticle-based lateral flow biosensor. This technology combines CPA determination with the restriction endonuclease cleavage of pollutants and side-flow biosensor analysis of the reaction products. Labeled CPA primers or probes are no longer used, and false-positive results generated by the interaction between the two modified CPA primers are effectively eliminated. The combination of these two primers with nucleic acid amplification methods has great advantages for accurate, rapid, sensitive, and simple target sequence detection. Huttunen et al. [152] developed a portable device based on a Raspberry Pi Zero W wireless single-board computer to measure fluorescent signals from a side-flow test paper. Zhong et al. [153] combined side-flow chromatography with an aptamer to develop new qualitative, semi-quantitative, and quantitative rapid detection techniques that are expected to be applied to the detection of GM food. Shuting et al. [154] constructed a general functional nucleic acid side-flow magnetic test strip using GM maize MON810 as the model target of the biosensor. This method does not rely on complex equipment, is fast and simple to operate, and meets the requirements for the rapid on-site screening of targets.

#### 4.3.2. Electrochemical Sensing Technology

Electrochemical DNA biosensors have high precision, good selectivity, economical operation, and efficient detection of specific DNA sequences in small samples. Currently, many DNA-based electrochemical sensors, including electrochemical biosensors [156], photoelectric chemical biosensors [157], electrochemical luminescence biosensors, electrochemical immunosensors [157], and electrochemical impedimetric biosensors [158] are widely used to detect transgenic components in plants. Cui et al. [159] developed a simple and easy-to-use electrochemical impedance (EI) gene sensor based on gold carbon dots (GCDs), composed of a carbon skeleton and gold nanoclusters, for the detection of GM maize. It consists of a handheld EI analyzer equipped with a coin-sized screen-printed carbon electrode modified with GCDs, and a fixed-capture probe via Au-S bonding for improved sensitivity and easy integration into portable devices for rapid analysis [157]. In addition, as a marker-free or hybridization indicator, DNA can be quantified by monitoring the change in electron transfer resistance during DNA hybridization, with a device that is simple in structure and convenient to operate.

#### 4.3.3. Other Types of Sensors 

DNA sensors usually have an oligonucleotide used as a probe, one end of which is combined with a suitable transducer. Quartz crystal microbalances (QCMs) and surface plasmon resonance (SPR) chips [160] are also commonly used.

The QCM is an acoustic sensor. A quartz crystal plate is used as an oscillator. When the plate oscillates regularly through an oscillating circuit, the adsorption of substances on the quartz plate decreases in frequency according to their mass [161]. Its working principle is to detect GMOs based on the mass increase caused by the hybridization of probes and unlabeled target DNA sequences. An ssDNA probe is fixed on the sensor surface of the QCM device, and hybridization between the fixed probe and the target complementary sequence in solution is monitored by its resonant frequency change. The DNA hybridization reaction in this detection technology does not require labeling (toxic compounds are not required) and the hybridization detection time is short. There are several studies on the detection of multiple targets using this technology [162,163,164]. While this technology, using coatings and surface modifiers in the form of functional nanoparticles, polymers, and complexes, has been widely explored [165], its development in GMO detection has been slow in recent years, possibly because of its delicate and complicated production process.

SPR is a powerful optical technique and one of the most attractive methods for surface-sensitive biomolecular interaction analysis and the real-time detection of various molecules [166]. It has the advantage of being able to analyze affinity and kinetics without labeling, and being able to directly use the characterization platform as a quantitative sensor. The basic principle of SPR is that the excitation of surface plasma depends on the refractive index of the medium surrounding the surface of the gold sensor attached to the ligand, which allows the detection of binding events through changes in the absorption and reflection of light [167]. Na et al. [168] developed a multi-selective, marker-free, renewable, and real-time GMO detection method based on an SPR platform. Typical genes of GM plant elements, *Tnos*, *CaMV 35S*, and *Cry1A*, were selected as targets, and the detection of the three genes was realized simultaneously, with a detection limit of 0.1 nM. Moreover, the hardware and software capabilities provided by smartphones can be integrated into SPR sensors to achieve more economical and accurate field-portable sensing for GMO detection [169].

## 5. Discussion and Prospects

With the continuous growth of the global population, the demand for food is also increasing. As the third-largest food crop in the world, maize is clearly important; therefore, the continuous improvement of maize traits is undoubtedly necessary. In 2022, Wenkang et al. [170] published the latest research results in *Science*, pointing out that exploring the role of the *KRN2*/*OsKRN2* gene in cereals may provide new opportunities to improve the yield of other global crops, such as maize. In recent years, advances in gene and genome sequencing technologies and the development of gene-editing technology have made precise and targeted editing possible [171,172]. The 2020 Nobel Prize in Chemistry was awarded to the inventors of CRISPR/Cas9 gene editing technology, which is easier, faster, cheaper, more precise, and safer than other technologies. The number of maize varieties that have undergone genetic modification using the CRISPR/Cas system [173] has been increasing in recent years [174,175]. It is precisely because of this that simultaneous efforts are needed to make the best use of the excellent new varieties of GM maize and to improve the regulatory means after their entry into the market to ensure the application of the required standards.

For nucleic acid detection methods, the efficiency and quality of nucleic acid extraction are the primary factors that determine the experimental effect. Appropriate extraction methods must be used for GM maize to meet different testing needs. The test objects can be divided into the raw materials of GM maize (leaves, seeds, endosperm, stems, and leaves) and its crude and highly processed products. Test application scenarios include laboratory settings and on-site tests in markets, fields, and shopping malls. Currently, no extraction method is applicable to all types of maize DNA. Therefore, it may be necessary to conduct comparative studies on emerging DNA extraction technologies based on different types of samples and different processing requirements in the future, to provide a research reference for the development of relevant detection methods. It must be stated that technologies based on MBs and microfluidic control may be of great help. MBs are small nanometers or particles, and their most useful property is their ability to achieve solid-phase reversible immobilization, which means that they can reversibly bind nucleic acids under dehydration conditions and, in the presence of strong magnets, MBs can be safely fixed during multiple washing and operating steps [71], facilitating nucleic acid extraction and purification. To a great extent, the traditional method (e.g., CTAB and SDS) eliminates the drawbacks of highly manual processes and equipment requirements and cumbersome operations, and the materials required are very cheap, which meet the requirements of commercial high-throughput testing. Technology combined with microfluidic control helps in the automation of extraction and purification, allowing precise control of the fluid within a very small margin of error, thereby accelerating and improving the flow of the sample preparation process and reducing reagent consumption, making the method suitable for point-of-care testing (POCT). Owing to their unique advantages, such as automation, miniaturization, and integration, microfluidic devices have been developed into a general tool in the fields of synthesis, detection, and analysis [176], which provides a feasible solution for the establishment of automated equipment integrated with multiplexed functional modules for DNA analysis. At present, many independent kits for automatic nucleic acid extraction are being developed [177,178], and there is reason to believe that they can also be used for nucleic acid extraction from GM maize.

For nucleic acid detection technology, the traditional amplification method has been able to achieve relatively accurate quantitative detection. However, it is still insufficient to meet the requirements of the accurate and rapid detection of multiple copies. The establishment of standards for real-time PCR will also focus on plasmid systems in the future. However, the increase in the variety of GM crops makes the construction of many standards more difficult. For isothermal amplification techniques, for which recombinant-enzyme-based assays are more suitable for commercial large-scale applications, the potential downside to their widespread use is the current patent situation. Since TwistDx moved to the USA, production delays, unavailability of TwistAmp nfo kit, and slow response from the technical team are the issues that may limit the utilization and adoption of RPA technology [179]. Gene chip technology has obvious advantages over these amplification methods, but it has some problems, such as a high cost and complicated technology, and its development has stagnated in recent years. Other emerging technologies, such as nucleic acid chromatography, may also have some problems. Although the device is simple and portable, there is a lot of room for development, and it is highly susceptible to interference and false-negative or false-positive results. The specific design of the detection instrument is complicated, and the detection time needs to be further optimized to shorten the assay time. Transgenic nucleic acid detection technology has three main developmental directions. First, existing traditional technologies need to be optimized. For example, Jia et al. [180] used an improved data classification method to develop a dPCR assay that can distinguish false-positive curves and improve the performance of low-concentration sample testing. The recognition error of the positive wells decreased by 64.4% compared to the typical static analysis technique. With these advantages, a real-time dPCR analyzer and an improved classification method can be used to enhance the performance of dPCR technology. The second direction is combined detection using multiple technologies, mainly the combination of new and traditional technologies, as well as combinations with biosensor technology, to realize the visualization of detection. Such combinations include an ultrasensitive test strip combined with the RPA and CRISPR/Cas12a systems for the rapid detection of GM crops [142,181]. Recombinant polymerase amplification combined with a label-free electrochemical impedance gene sensor has been used to detect GM maize [159]. The third direction is the development of new technologies, building new detection systems in principle and form, and changing and discovering new available materials, which may require long-term research. Liu et al. [182] constructed a rapid and ultrasensitive fluorescence-sensing platform for *CaMV 88S* promoter detection based on facile fluorescent probes and nanoscale Fe-MIL-35. The use of an enzyme-free method reduces the detection cost, and the sensor manufacturing process is simple and can be used for commercial applications. Thus, improving the efficiency and accuracy of detection remains a challenge for the development of GMO detection systems. To meet the detection requirements of different settings, the operation of these methods will need to be more convenient and the equipment will need to be smaller and less expensive. With recent developments in artificial intelligence technology, the automation of the detection process is gradually being considered by researchers in the field. A long and tireless effort will be required to provide robust safety guarantees for agricultural practices involving GM crops.

## Figures and Tables

**Table 1:** Presents partial information on the GM maize varieties approved for use worldwide.

Transformer Name	Characters	Research and Development Institutions	Target Genes	The Earliest Approved Use Time
PY203	Quality improvement	Agriveda	*PHY02*, *pmi*	2021
DP202216	Herbicide resistance, increased production	Dow AgroSciences LLC	*zmm28*, *mo-pat*	2020
DBN9858	Glyphosate herbicide tolerance, glyphosate herbicide tolerance	Beijing Dabeinong	*EPSPS (Ag)*, *pat*	2020
DBN9936	lepidopteran pests, herbicide-tolerant	Beijing Dabeinong	*Cry1Ab*, *EPSPS*	2019
MZIR098	Resistance to lepidopteran pests, herbicide-tolerant	Syngenta	*eCry3.1Ab*, *mCry3A*, *pat*	2016
MON87403	Increase production	Monsanto	*ATHB17*	2015
5307	Resistant to lepidoptera pests	Syngenta	*eCry3.1Ab*	2013
4114	Resistance to lepidoptera and coleoptera pests, herbicide resistance	DuPont	*Cry1F*, *Cry34Ab1*, *Cry35Ab1*, *pat*	2013
DAS40278-9	Herbarium resistant	Dow AgroSciences LLC	*aad-1*	2012
DP32138-1	Male sterile	DuPont	*Ms45*, *zm-aa1*	2011
MON87460	Fight a drought	Monsanto	*CspB*	2010
MIR604	Antilepidopteran pests	Syngenta	*mCry3A*	2007
680	Male sterility, herbicide-tolerant	DuPont	*pat*, *DAM*	1998
Bt176	lepidopteran pests, herbicide-tolerant	Syngenta	*Cry1Ab,* *bar*	1995
DBN9936	lepidopteran pests, herbicide-tolerant	Beijing Dabeinong	*Cry1Ab*, *EPSPS*	2019
MZIR098	Resistance to lepidopteran pests, herbicide-tolerant	Syngenta	*eCry3.1Ab, mCry3A,* *pat*	2016
MON87403	Increase production	Monsanto	*ATHB17*	2015
5307	Resistant to lepidoptera pests	Syngenta	*eCry3.1Ab*	2013
4114	Resistance to lepidoptera and coleoptera pests, herbicide resistance	DuPont	*Cry1F, Cry34Ab1*, *Cry35Ab1, pat*	2013
DAS40278-9	Herbarium resistant	Dow AgroSciences LLC	*aad-1*	2012
DP32138-1	Male sterile	DuPont	*Ms45, zm-aa1*	2011
MON87460	Fight a drought	Monsanto	*CspB*	2010
MIR604	Antilepidopteran pests	Syngenta	*mCry3A*	2007
680	Male sterility, herbicide-tolerant	DuPont	*pat*, *DAM*	1998
Bt176	lepidopteran pests, herbicide-tolerant	Syngenta	*Cry1Ab*, *bar*	1995

## Data Availability

The data presented in this study are available in the article.
